# Erythropoietin suppresses osteoblast apoptosis and ameliorates steroid-induced necrosis of the femoral head in rats by inhibition of STAT1-caspase 3 signaling pathway

**DOI:** 10.1186/s12891-023-07028-y

**Published:** 2023-11-17

**Authors:** Tingwen Cai, Siyuan Chen, Chenghu Wu, Chao Lou, Weidan Wang, Chihao Lin, Hongyi Jiang, Xinxian Xu

**Affiliations:** 1 Department of Orthopedics, The Second Affiliated Hospital, Yuying Children’s Hospital of Wenzhou Medical University, Wenzhou, Zhejiang Province China; 2 Key Laboratory of Orthopedics of Zhejiang Province, Wenzhou, Zhejiang Province China; 3 The Second School of Medicine, Wenzhou Medical University, Wenzhou, Zhejiang Province China; 4 School of Biomedical Engineering, School of Ophthalmology and Optometry and Eye Hospital, Wenzhou Medical University, Wenzhou, Zhejiang Province China

**Keywords:** Erythropoietin, Apoptosis, Necrosis of the femoral head, STAT1-caspase 3

## Abstract

**Background:**

Steroid-induced avascular necrosis of the femoral head (SANFH) is characterized by osteoblast apoptosis, leading to a loss of bone structure and impaired hip joint function. It has been demonstrated that erythropoietin (EPO) performs a number of biological roles.

**Objective:**

We examined the effects of EPO on SANFH and its regulation of the STAT1-caspase 3 signaling pathway.

**Method:**

In vitro, osteoblasts were treated with dexamethasone (Dex) or EPO. We identified the cytotoxicity of EPO by CCK-8, the protein expression of P-STAT1, cleaved-caspase9, cleaved-caspase3, Bcl-2, BAX, and cytochrome c by Western blotting, and evaluated the apoptosis of osteoblasts by flow cytometry. In vivo, we analyzed the protective effect of EPO against SANFH by hematoxylin and eosin (H&E), Immunohistochemical staining, and Micro-computed tomography (CT).

**Results:**

In vitro, EPO had no apparent toxic effect on osteoblasts. In Dex-stimulated cells, EPO therapy lowered the protein expression of BAX, cytochrome c, p-STAT1, cleaved-caspase9, and cleaved-caspase3 while increasing the expression of Bcl-2. EPO can alleviate the apoptosis induced by Dex. In vivo, EPO can lower the percentage of empty bone lacunae in SANFH rats.

**Conclusion:**

The present study shows that EPO conferred beneficial effects in rats with SANFH by inhibiting STAT1-caspase 3 signaling, suggesting that EPO may be developed as a treatment for SANFH.

**Supplementary Information:**

The online version contains supplementary material available at 10.1186/s12891-023-07028-y.

## Introduction

Prolonged and high-dose steroid administration commonly leads to the development of steroid-induced avascular necrosis of the femoral head (SANFH), which can result in a significant disability rate [1]. Left untreated, SANFH can lead to a collapse of bone structure and impaired hip joint function, eventually requiring surgical intervention. This progressive deterioration can have significant psychological and financial costs for patients and society [2]. An excessive amount of corticosteroids can lead to a reduction in the lifespan of osteoclasts, the induction of cell death in osteoblasts, endothelial cells, and osteocytes, as well as the inhibition of osteoblast and osteoclast development [3]. An excessive amount of corticosteroids can lead to a reduction in the lifespan of osteoclasts, the induction of cell death in osteoblasts, endothelial cells, and osteocytes, as well as the inhibition of osteoblast and osteoclast development [4]. As a kind of skeletal component cell, osteoblasts contribute to the pathogenesis of non-traumatic osteonecrosis of the femoral head and bone growth, maintenance, and repair [5]. In addition, it is reported that dexamethasone (Dex) can induce apoptosis of rat osteoblasts [6, 7].

Signal transducer and activator of transcription (STAT) is a family of cytoplasmic proteins that can bind to DNA in the regulatory region of target genes. Once activated, it can regulate the expression of many apoptosis-related genes, cell growth, and immunology [8, 9]. STAT1 has two phosphorylation sites, including tyrosine phosphorylation, and serine phosphorylation, which can promote apoptosis when activated [10, 11]. Caspase-3, one of the most essential executors of apoptosis, can spontaneously regulate cell life activities. Moreover, its activation (usually a slow process) will execute the process of cell apoptosis [12]. Additionally, prior research has demonstrated that the STAT1/caspase3 pathway is crucial for the developing of SANFH [13, 14].

Erythropoietin (EPO) is the first hematopoietic growth factor found and has clinical application value. EPO has the effects of anti-inflammation, anti-oxidation, anti-apoptosis, and improving local microcirculation [15–18]. Further, EPO can contribute to the process of hematopoiesis by controlling the expression of STAT1 and STAT3, according to research by Kirito K et al. [19]. Liang C et al. have found that EPO can reduce the degree of cerebral ischemia injury by down-regulating the expression of p-STAT1 and upregulating the expression of p-STAT3 and reducing the number of brain cell apoptosis [20]. Furthermore, EPO can down-regulate Caspase3 and upregulate the expression of vascular endothelial growth factor (VEGF) and delay the development of prevention of SANFH by reducing the apoptosis rate of osteoblasts and osteoclasts [21]. Additionally, a composite stent loaded with EPO benefits the repair of SANFH by enhancing the osteogenic ability and promoting the expression of angiogenic factors [22]. While the therapeutic potential of EPO in treating SANFH has been established, its specific impact on dexamethasone-induced osteoblast apoptosis remains unclear. Therefore, this study aimed to investigate the effect of EPO on dexamethasone-induced osteoblast apoptosis, both in vitro and in an SANFH animal model, and explore the underlying mechanisms.

## Materials and methods

### Ethics statement and experimental animals

All experimental procedures were by the recommendations in the Guide of the Care and Use of Experimental Animals laid down by the National Institutes of Health and were approved by the Committee for Animal Experimentation of Wenzhou Medical University.

### Reagents and antibodies

Recombinant human EPO (PeproTech, USA) was dissolved in 0.1% Bovine serum albumin (BSA) (Sigma, MO, USA). Type I collagenase, dexamethasone (Dex), and dimethylsulfoxide (DMSO)were purchased from Sigma Chemical Co. (MO, USA). Antibodies against STAT1, caspase 3, Cleaved-caspase 9, caspase 9, BAX, Bcl-2, and GAPDH were obtained from Proteintech (Wuhan, China). Goat anti-rabbit and anti-mouse IgG-HRP were obtained from Biosharp Life Sciences (Anhui, China), and primary antibodies against Cleaved-caspase3 were acquired from Cell Signaling Technology (MA, USA). Dojindo (Kumamo, Japan) provided the Cell-Counting Kit-8 (CCK-8). Primary antibodies directed against P-STAT1, Cytochrome C, and Alexa Fluor®488-labeled goat anti-rabbit IgG (H + L) secondary antibodies were obtained from Abcam (Cambridge, MA, USA). Meilunbio (Dalian, China) provided the annexin V-FITC/propidium iodide (PI) double staining.

### Cell culture

The cranium of newborn rats (within 10 days of birth; Animal Center of the Chinese Academy of Sciences, Shanghai, China) was aseptically removed following euthanasia with pentobarbital sodium. The extracted skull tissue was soaked in sterile phosphate-buffered saline (PBS). Connective tissue was carefully separated, and the skull tissue was cut into small pieces measuring 1 × 1 mm^2. Subsequently, the tissue was washed thrice times with PBS and digested for 20 min using a 0.25% trypsin-EDTA solution. Subsequently, the tissue was washed thrice with PBS and digested for 20 min using a 0.25% trypsin-EDTA solution. Subsequently, the tissue was incubated with 0.2% collagenase in a CO2 incubator (5%) at 37 °C for 1 h. The digested tissue solution was centrifuged at 1000 rpm for 5 min, and the supernatant was discarded. The resulting osteoblasts were suspended in DMEM containing 1% penicillin/streptomycin and 10% FBS. Osteoblasts were identified and passaged at 80-90% confluency using a 0.25% trypsin-EDTA solution. For this study, only 0 to 2 passages of cells were used to ensure phenotypic stability.

### Cell survival

Osteoblasts were treated with different concentrations of EPO (0, 10, 50, 100 IU/ml) for 24 h, and in some groups, the cells were co-cultured with Dex (1µM) for 24 h. The cell count kit (CCK-8, Dojindo, Japan) was used to measure the survival rate of osteoblasts according to the manufacturer’s instructions. Record each well’s CCK-8 optic density (OD) at 450 nm using a spectrophotometer.

### LDH release assay

The cytotoxicity of Dex was detected by the lactate dehydrogenase (LDH) release method. Osteoblasts were treated with Dex (1µM) and then incubated at 37 ℃ in a 5% CO2 incubator with different concentrations of EPO (0, 10, 50, 100 IU/ml) for 24 h. According to the manufacturer’s instructions, samples of the cell culture media from each well were taken, and the LDH activity was measured (Solarbio, Beijing, China).

### Western blot analysis

Osteoblasts were treated with Dex (1µM) for 24 h. In some experiments, cells were co-treated with EPO (50 IU/ml) or pre-treated with the STAT1 inhibitor fludarabine (100µM) for 1 h, followed by the treatment of Dex. Both adhering and floating cells were gathered at the end of the experiment. Osteoblast proteins were extracted using radioimmunoprecipitation (RIPA) buffer containing 1% phenylmethane sulfonyl fluoride (PMSF) and 1% phosphatase inhibitor cocktail (100X). A BCA protein assay kit (Beyotime) measured the total protein content. After measurement, A total of 30 µg of protein, loaded into each well of the gel, had been resolved by 10% sodium dodecyl sulfate-polyacrylamide gel electrophoresis were transferred to room temperature for 30 min using a polyvinylidene fluoride membrane (Millipore, Billerica, MA, USA). After blocking with 5% non-fat milk solution for 60 min, the membrane was incubated with the following primary antibodies: anti-P-STAT1, anti-STAT1, anti-Cleaved-caspase 3, anti-caspase 3, anti-Cleaved-caspase 9, anti-caspase 9, anti-Cytochrome C, anti-BAX, and anti-Bcl-2. After an overnight stay at 4 °C of incubation, membranes were then exposed for two hours to the secondary antibody. Proteins were detected using ECL detection kits from Beyotime, and their quantities were determined using Image Lab 3.0 (Bio-Rad, Hercules, CA, USA).

### Immunofluorescence staining

Osteoblasts were cultured on glass slides in a 12-well plate and incubated overnight in conditioned media. The cells were then treated with Dex (1µM) for 24 h. In some groups, osteoblasts were co-treated with EPO (50 IU/ml) for an additional 24 h. After treatment, the samples were rinsed three times with PBS and fixed with 4% paraformaldehyde for 15 min. Permeabilization was performed using 0.5% Triton X-100 (SolarBio) for 5 min. To block non-specific binding, a 30-minute incubation with 10% goat serum at room temperature was carried out. Subsequently, cells were incubated overnight at 4 °C with rabbit anti-P-STAT1 or rabbit anti-cleaved-caspase 3 antibodies. Following antibody incubation, cells were stained with goat anti-rabbit IgG (Alexa Fluor 488) (Abcam). After three washes with PBS, all cells were counterstained with DAPI (Beyotime) and observed using a laser scanning confocal microscope (Olympus). The fold increase relative to the baseline level of the control group was used to express the results.

### Flow cytometric analysis

Apoptotic osteoblasts were assessed using flow cytometric analysis. Osteoblasts were seeded in 12-well plates and stimulated with or without Dex (1µM) for 24 h. In certain experiments, osteoblasts were co-cultured with EPO (50IU/ml) for 24 h. Following collection, the cells were washed twice with PBS and resuspended in 100 µl of 1X Annexin V binding buffer. Subsequently, 5 µl of FITC-Annexin V was added, and the cells were incubated for 15 min at room temperature in the absence of light. The apoptosis rate of osteoblasts was then measured using flow cytometry (BD Biosciences, USA).

### Model of SANFH

A total of 30 mature male Sprague-Dawley (SD) rats weighing between 180 and 200 g were acquired from the Chinese Academy of Sciences’ central animal house (Shanghai, China). All rats were kept in specially made cages with full access to food and water in normal circumstances (temperature 22 ± 2 °C and humidity 50 ± 5%). The animals were randomly allocated to three groups (n = 10 rats/group) using a random number table as follows: control group (intramuscular administration of a sham injection), Dex group (intramuscular administration of Dex at 10 mg/kg), and Dex + EPO group (treatment with EPO (500 U/kg/day) after the administration of Dex (10 mg/kg)]. Dex and the sham injection with normal saline (NS) were administered to rats thrice weekly for 8 continuous weeks. EPO was administered thrice weekly starting 1 week following the initial injection of Dex.

### Micro-computed tomography (CT) and quantitative analysis

Eight weeks after the last Dex injection, the rat femoral head microstructures were measured by a micro-CT scan. Before analysis, femur samples were collected and stored in 4% paraformaldehyde. Specimens were scanned on a Micro-CT system (70 kV, 114 A; micro-CT 80 scanner; Scanco Medical, Bassersdorf, Switzerland). A workstation for analysis was used to rebuild the three-dimensional (3D) digital pictures. The diagnostic criteria for femoral head necrosis are as follows: trabecular bone fracture, cystic change, hardened zone of femoral head, or flat. The proximal femur’s relative bone mass was calculated using measurements of bone mineral density (BMD), trabecular thickness (Tb. Th), trabecular separation (Tb. Sp), bone volume per tissue volume (BV/TV), and trabecular number (Tb. N) [23, 24].

### Histological examination

Paraffin sections from each rat were deparaffinized with xylene and then rehydrated in a graded ethanol series. Following a deionized water wash, the rehydrated sections were stained for 5 min with 2% hematoxylin and 1 min with 2% eosin. All sections were assessed under the microscope (Olympus, Japan). The presence of empty lacunae was assessed across each section. Based on the ratio of empty lacunae, as previously stated, osteonecrosis was primarily identified [14].

### Immunohistochemical analysis

A range of ethanol concentrations was used to hydrate paraffin-embedded 5 mm tissue slices after dewaxing them with xylene. Sections were then covered with endogenous peroxidase blocking agent (ZSGB-BIO, Beijing, China) for 15 min, then immersed in sodium citrate solution for antigen repair, then blocked with 10% goat blocking serum for 30 min at room temperature, then incubated overnight with Cleaved-caspase3 or P-STAT1 primary antibody (1:200) at 4℃, and then incubated with HRP-conjugated secondary antibody and diamino benzene (DAB) (ZSGB-BIO, Beijing, China). Sections were kept at 4 °C.

### TdT-mediated dUTP nick-end labeling (TUNEL)

Osteoblast apoptosis was assessed using the DeadEndTM Fluorometric TUNEL System (Promega, Madison, Wisconsin, USA). Following the manufacturer’s instructions, the cells on the slides were fixed and made permeable before being treated with the cell death detection kit’s chemicals. DAPI-containing aqueous mounting solution was used to stain the nuclei (Santa Cruz, USA). Lastly, pictures were taken using an inverted fluorescent microscope (Olympus, Tokyo, Japan).

### Statistical analysis

Data are expressed as mean ± SD. Statistical analyses employed SPSS 20.0, using one-way analysis of variance (ANOVA) and Tukey’s test to compare treated and untreated cells and tissues. P values < 0.05 were significant.

## Results

### The protective effect of EPO on osteoblasts under Dex stimulation

**Fig. 1:**
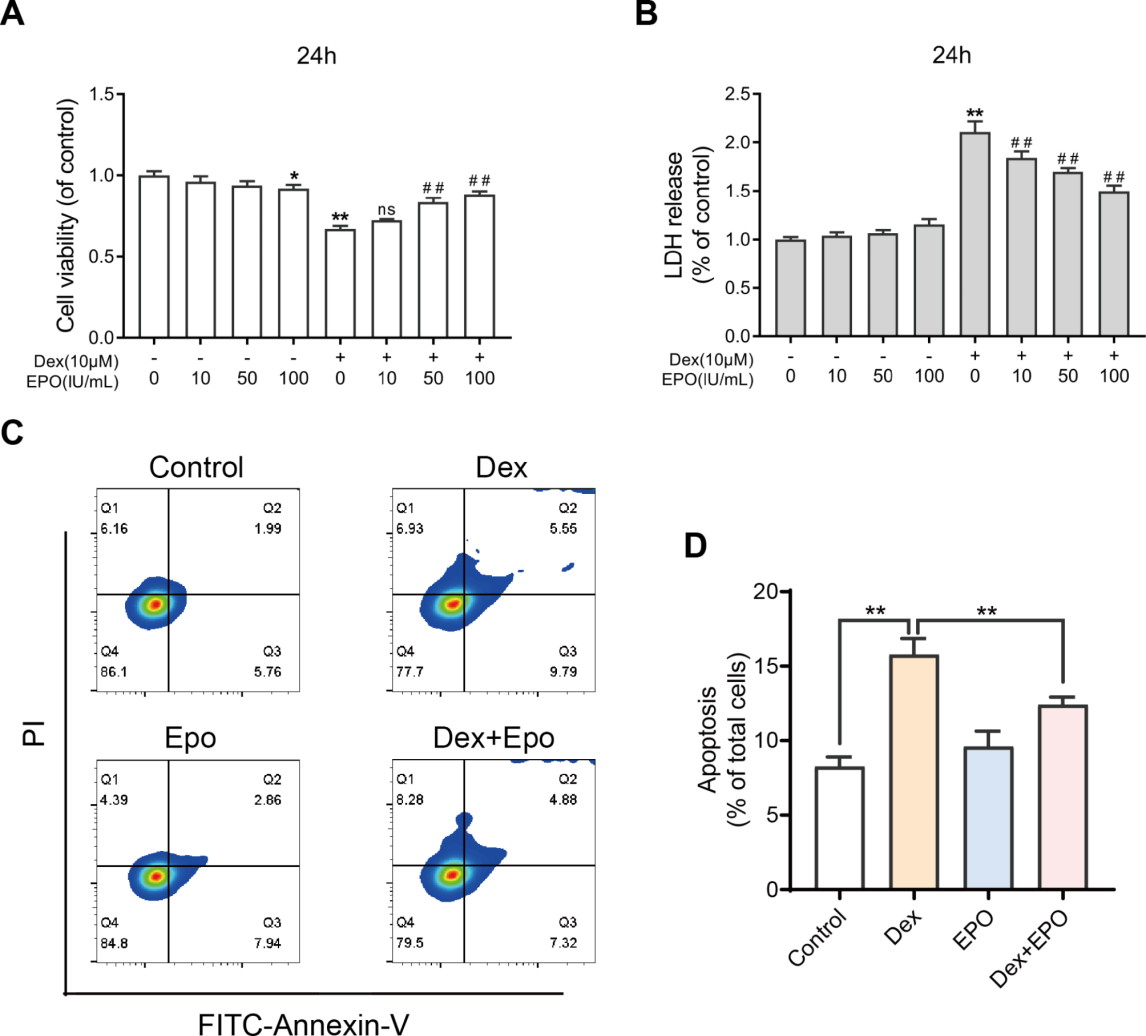
The protective effect of EPO on osteoblasts under Dex stimulation. CCK8 assay was used to determine the cytotoxicity of osteoblasts treated with EPO at different concentrations (10 IU/ml, 50 IU/ml, 100 IU/ml) or co-treated with Dex for 24 h **(A)**. The protective effect of EPO against Dex-induced cell damage was observed by LDH release **(B)**. Values represent the averages ± S.D. Significant differences between different groups are indicated as *P < 0.05, **P < 0.01, vs. control group; #P < 0.05, ##P < 0.01, vs. Dex alone treatment group, n = 5. The apoptosis of osteoblasts induced by Dex together with or without EPO were assessed through Annexin V-FITC/PI double staining with flow cytometric analysis **(C, D**). Values represent the averages ± S.D. Significant differences between different groups are indicated as *P < 0.05, **P < 0.01, n = 3

Osteoblasts were treated with Dex (1µM) and various concentrations of EPO (10 IU/ml, 50 IU/ml, 100 IU/ml). As shown in Fig. 1A and B, EPO (from a concentration of 10 IU/ml to 50 IU/ml) shows no significant toxicity to osteoblasts. However, there was a statistically significant decrease in cell viability at 100 IU/ml. Meanwhile, Dex caused apoptosis, drastically decreasing the number of still-alive osteoblasts, and EPO can repair this harm in a concentration-dependent way. Alternatively, Dex can increase LDH release in osteoblasts, which EPO reverses. To replicate osteoblast apoptosis, Dex was utilized as a model to examine the impact of EPO. Flow cytometry results showed that Dex could induce apoptosis in osteoblasts, while EPO had significant anti-apoptotic effects, and that treatment of cells with EPO alone did not increase osteoblasts apoptosis (Fig. 1C and D).

### Effects of EPO on STAT1/caspase3 pathway in osteoblasts treated with Dex

**Fig. 2:**
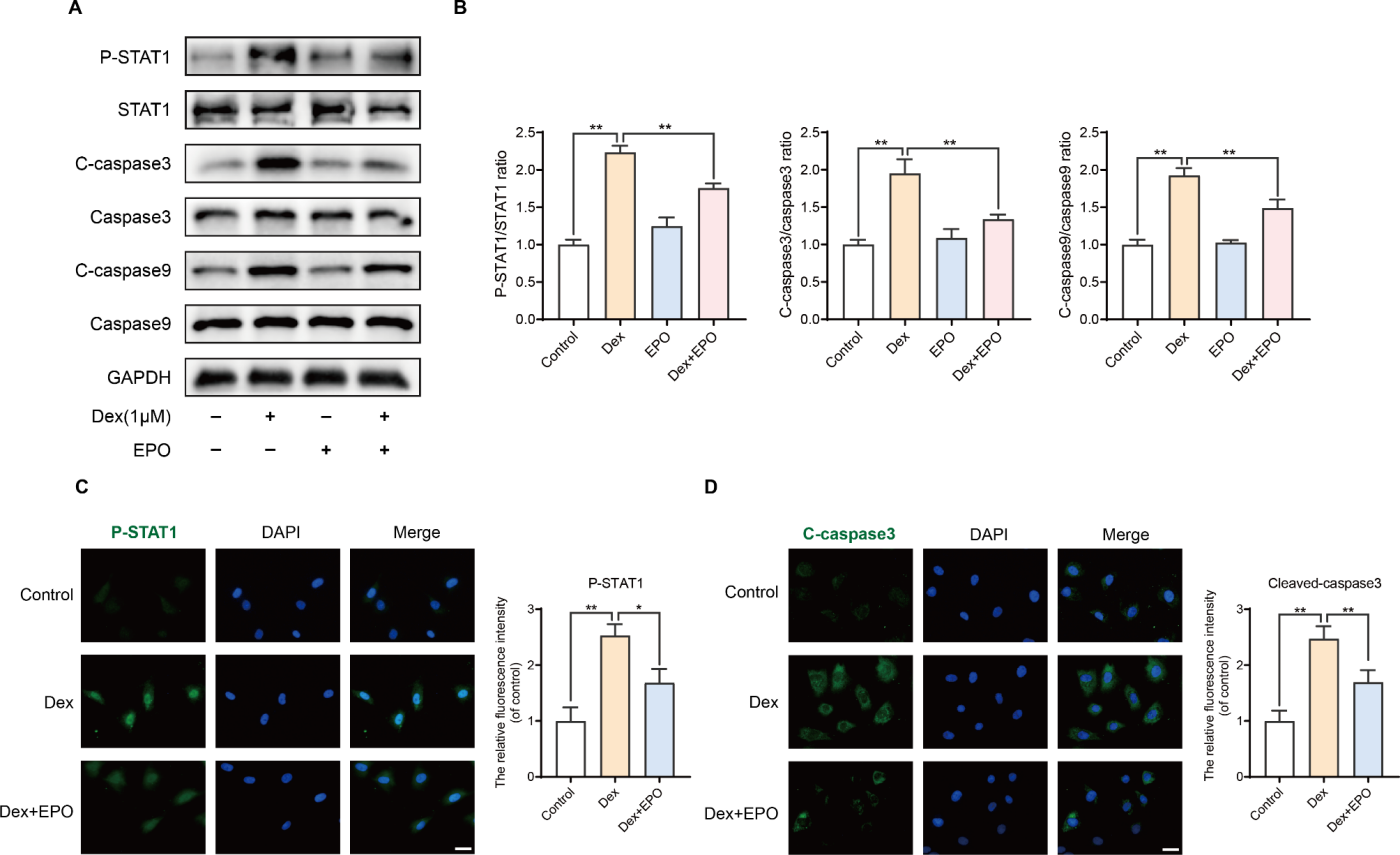
Effects of EPO on STAT1/caspase3 pathway in osteoblasts treated with Dex. The protein expression of P-STAT1, STAT1, cleaved-caspase3, caspase3, cleaved-caspase9 and caspase9 were measured by western blot **(A)** and quantification of the resulting bands **(B)**. Values represent the averages ± S.D. Significant differences between different groups are indicated as *P < 0.05, **P < 0.01, n = 3. Osteoblasts were stimulated with Dex or co-cultured with EPO for 24 h. Typical P-STAT1 **(C)** and cleaved caspase-3 **(D)** were detected by immunofluorescence combined with DAPI staining for nuclei (scale bar: 10 μm). Values represent the averages ± S.D. Significant differences between different groups are indicated as *P < 0.05, **P < 0.01, n = 3

Western blotting was utilized to assess the expression levels of STAT1, P-STAT1, caspase3, cleaved-caspase3, caspase9, and cleaved-caspase9 in osteoblasts, aiming to investigate the impact of EPO on the STAT1/caspase3 pathway in these cells. Following 24 h of dexamethasone stimulation, there was a significant increase in P-STAT1, Cleaved-caspase3, and Cleaved-caspase9 levels. However, co-treatment of osteoblasts with EPO inhibited the dexamethasone-induced upregulation of P-STAT1, Cleaved-caspase3, and Cleaved-caspase9 (Fig. 2A and B). To further explore the effect of STAT1 on caspase-3 expression, osteoblasts were stimulated with Dex (1µM) for 24 h and co-treated with or without EPO (50 IU/ml). Immunofluorescence analysis revealed that nuclear expression of P-STAT1 was minimal in control osteoblasts, while cytoplasmic expression of Cleaved-caspase3 was also minimal. However, their expression significantly increased after Dex induction. Furthermore, EPO inhibited the Dex-induced expression of Cleaved-caspase3 and P-STAT1 (Fig. 2C and D), further confirming that EPO hinders Dex-induced apoptosis via the STAT1/caspase3 signaling pathway.

### EPO’s attenuation of osteoblast apoptosis via the mitochondrial pathway

**Fig. 3:**
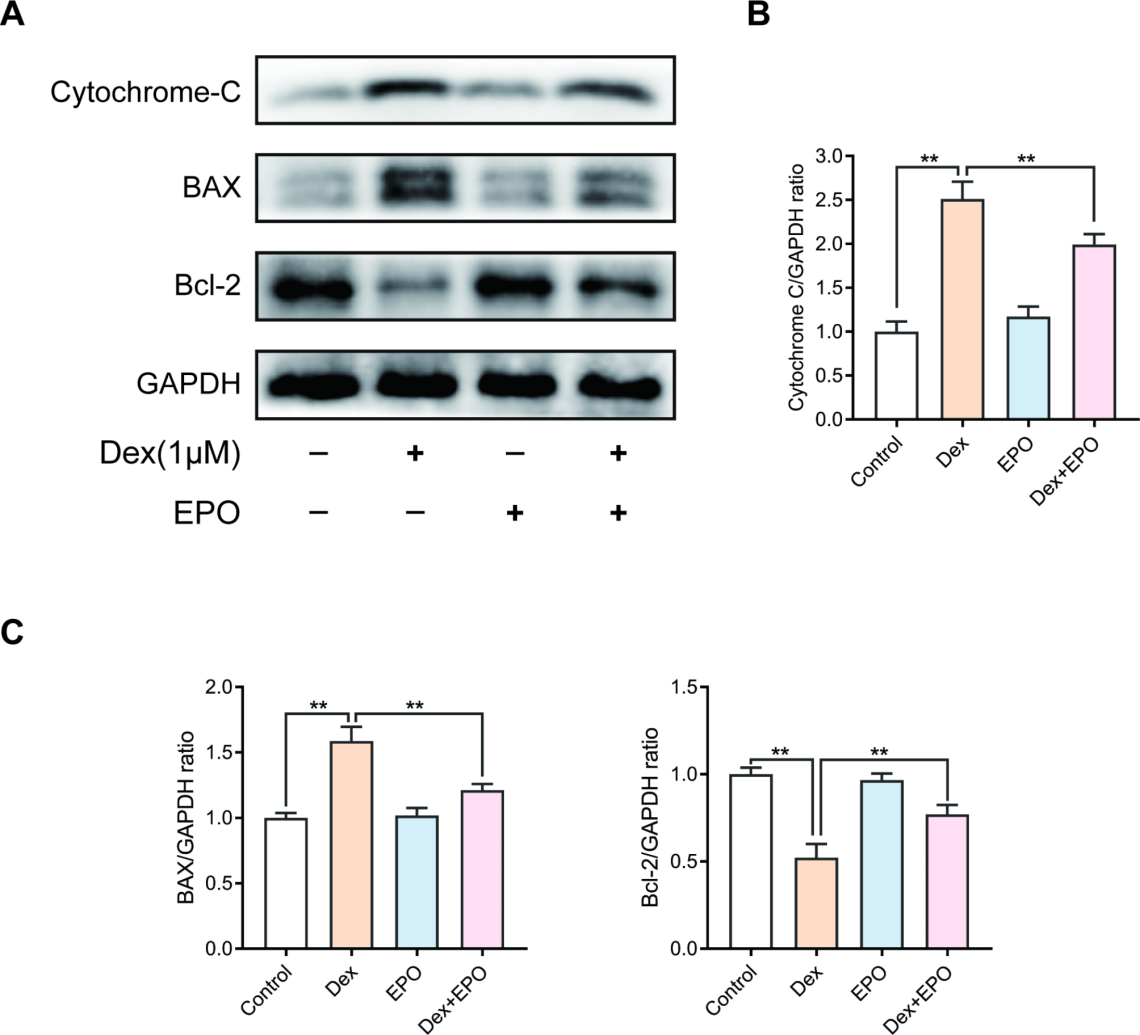
EPO’s attenuation of osteoblast apoptosis via the mitochondrial pathway. The protein expressions of Bcl-2, BAX and cytochrome c in osteoblasts treated above were detected by Western blotting **(A)** and quantification of the resulting bands **(B)**. Values represent the averages ± S.D. Significant differences between different groups are indicated as *P < 0.05, **P < 0.01, n = 3

In order to determine if the mitochondrial pathway is involved in the anti-apoptotic effect caused by EPO, the expression changes of the proteins linked to the mitochondrial route were examined using Western blotting. As seen in Fig. 3, BAX and cytochrome C protein expression levels dramatically rose following Dex stimulation, but this trend was suppressed in the group that received EPO. Bcl2’s expression alterations were the antithesis of those seen in BAX and cytochrome C.

### Effect of EPO and fludarabine on the STAT1/caspase3 pathway in Dex-treated osteoblasts

**Fig. 4:**
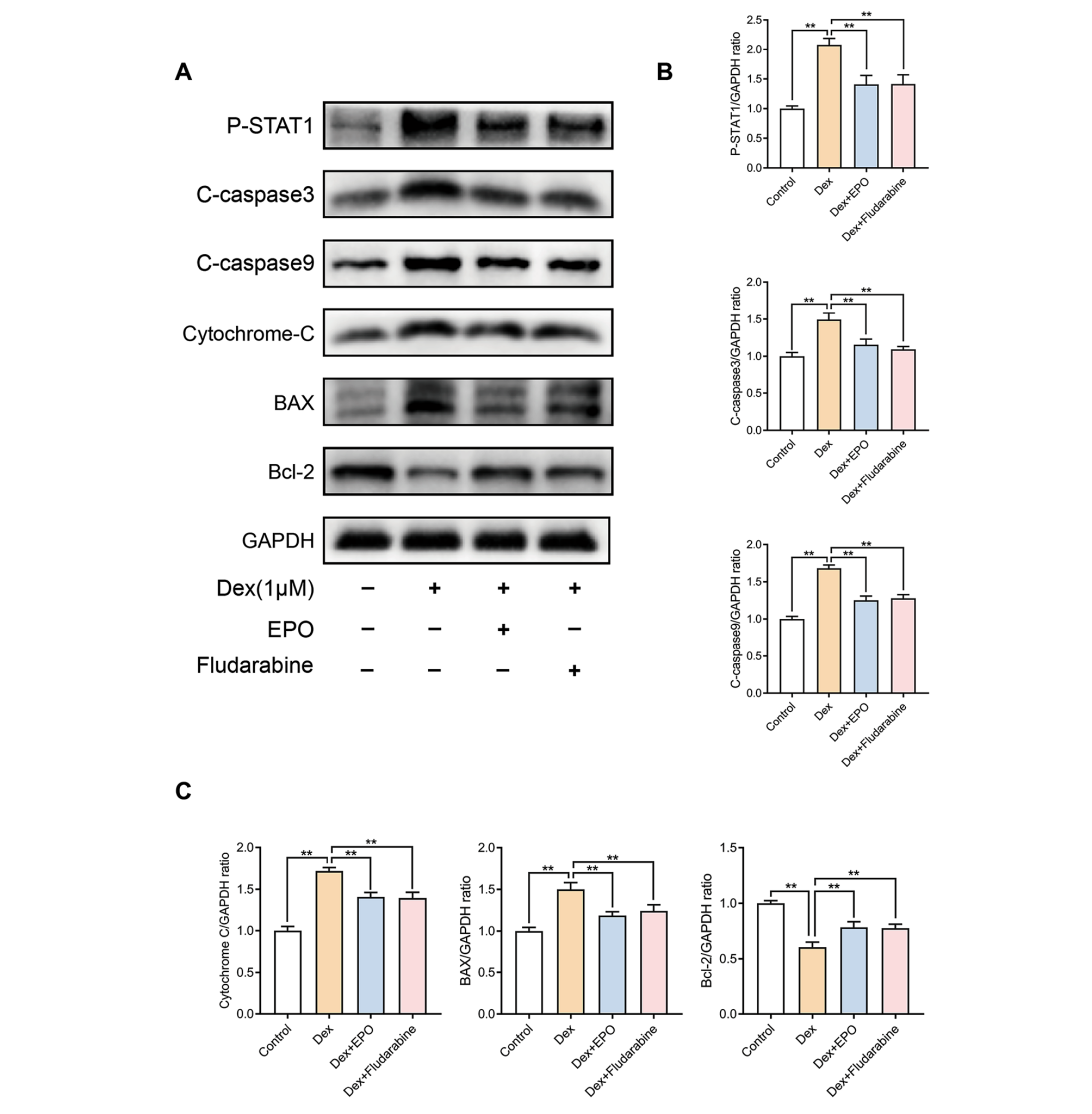
Effect of EPO and fludarabine on the STAT1/caspase3 pathway in Dex-treated osteoblasts. As described above, the osteoblasts were treated with Dex, EPO, or Fludarabine. The protein expression of P-STAT1, cleaved-caspase3, cleaved-caspase9, Bcl-2, BAX and cytochrome c were measured by western blot **(A)** and quantification of the resulting bands **(B, C)**. Values represent the averages ± S.D. Significant differences between different groups are indicated as *P < 0.05, **P < 0.01, n = 3

To further explore the effect of EPO on the STAT1/caspase3 pathway in osteoblasts, we again treated the cells induced by Dex with EPO and Fludarabine, respectively. As shown in Fig. 4, both fludarabine and EPO reversed the Dex-induced changes in the expression levels of P-STAT1, Cleaved-caspase3, Cleaved-caspase9, BAX, cytochrome C, and Bcl-2. It was further suggested that EPO can regulate the STAT1/caspase3 pathway in osteoblasts.

### EPO alleviated steroid-induced avascular necrosis of the femoral head (SANFH)

**Fig. 5:**
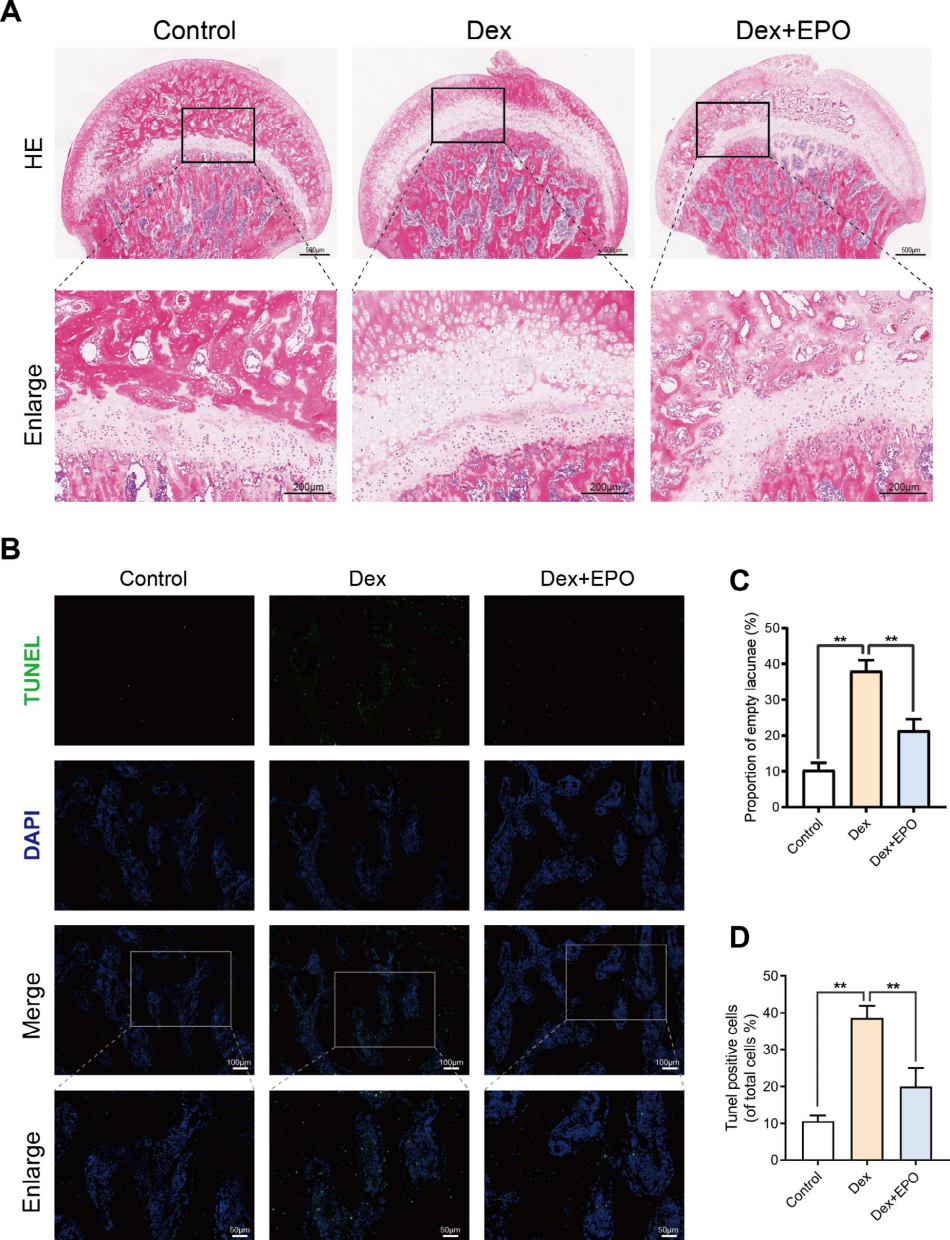
EPO alleviated steroid-induced avascular necrosis of the femoral head (SANFH). The control group’s femoral head included no empty lacunae. While there were few empty bone lacunae in the Dex + EPO group, the Dex group displayed a significant number of empty bone lacunae and necrotic bone marrow cells **(A, C)**. Representative images of TUNEL staining of the sections (scale bar: 100 μm and 50 μm) **(B)** and quantitative analysis of TUNEL staining **(D)**. Values represent the averages ± S.D. Significant differences between different groups are indicated as *P < 0.05, **P < 0.01, n = 5

In the femoral head, SANFH is characterized by multiple pyknotic nuclei of osteocytes and thin, sparse bone trabeculae with broad, empty lacunae. Here, we defined osteonecrosis primarily based on the ratio of empty lacunae. Significantly more rats in the Dex group (9 of 10) developed osteonecrosis compared to only 5 in the EPO + Dex group (Fig. 5A). In contrast, no rats in the control developed osteonecrosis. The ratio of empty lacunae was significantly higher in the Dex group when compared to the controls and significantly lower in the EPO + Dex group when compared to the Dex group, indicating that EPO may suppress the formation of SANFH in vivo (Fig. 5C). Furthermore, EPO treatment significantly reduced the number of TUNEL-positive cells in SANFH (Fig. 5B, D).

### EPO inhibited the expression of P-STAT1 and cleaved-caspase3 in steroid-induced avascular necrosis of the femoral head (SANFH)

**Fig. 6:**
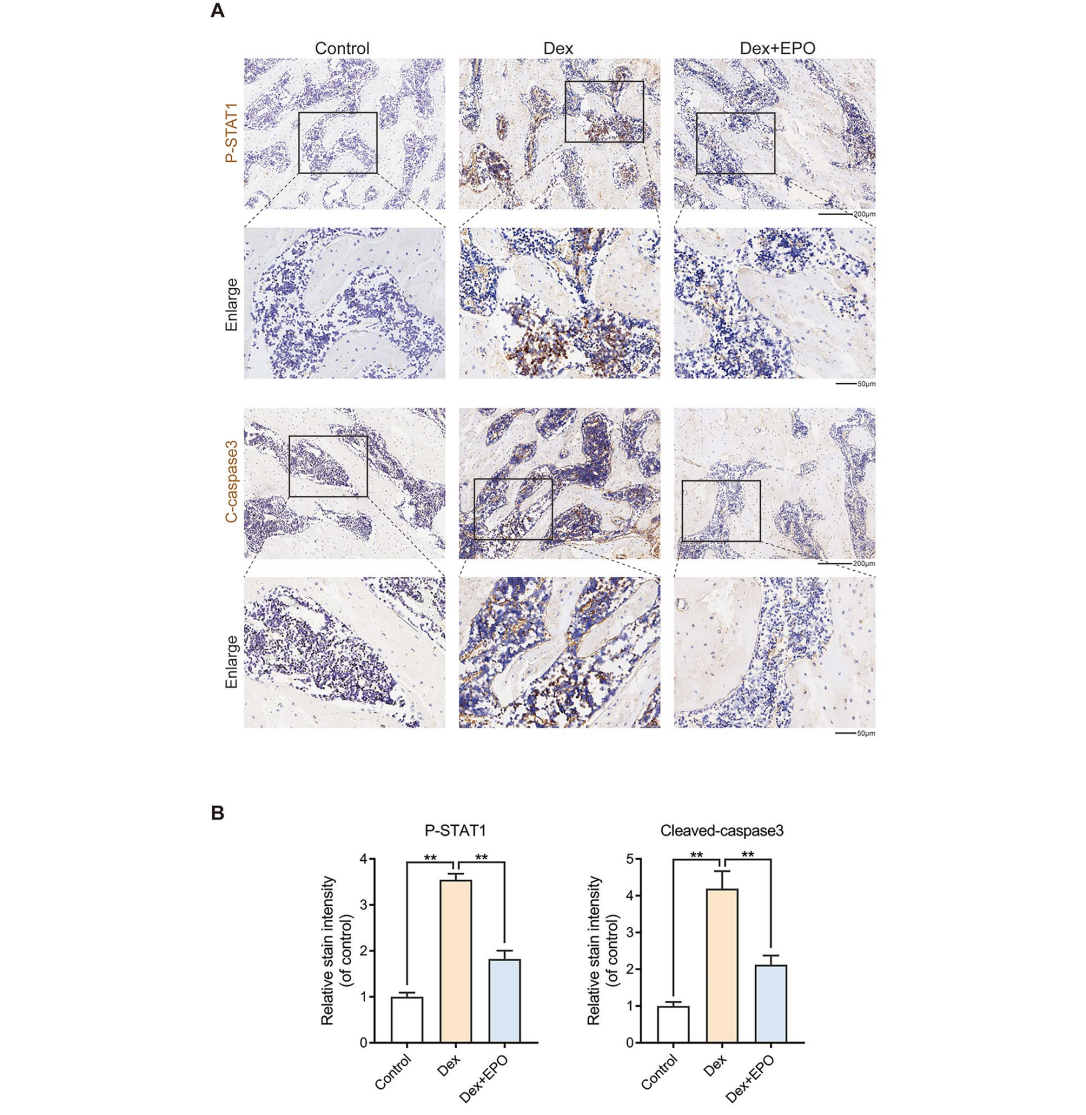
EPO inhibited the expression of P-STAT1 and cleaved-caspase3 in steroid-induced avascular necrosis of the femoral head (SANFH). Activation of caspase3 and STAT1 is involved in apoptotic cell death. Positive expressions were found in the Dex groups **(A)**. The percentages of P-STAT1 and Cleaved-caspase3 positive cells in each section were quantified by Image J software **(B)**. Values represent the averages ± S.D. Significant differences between different groups are indicated as *P < 0.05, **P < 0.01, n = 5

The immunohistochemistry test was used to measure the amounts of Cleaved-caspase3 and P-STAT1 protein expression to examine EPO’s impact on those proteins in vivo. In the control group, there was hardly any positive expression at all. The Dex group, however, had a more significant percentage of Cleaved-caspase-3, P-STAT1-positive cells, and EPO might counteract the effects of DEX mentioned above (Fig. 6).

### EPO prevented bone loss in the SANFH model of rats

**Fig. 7:**
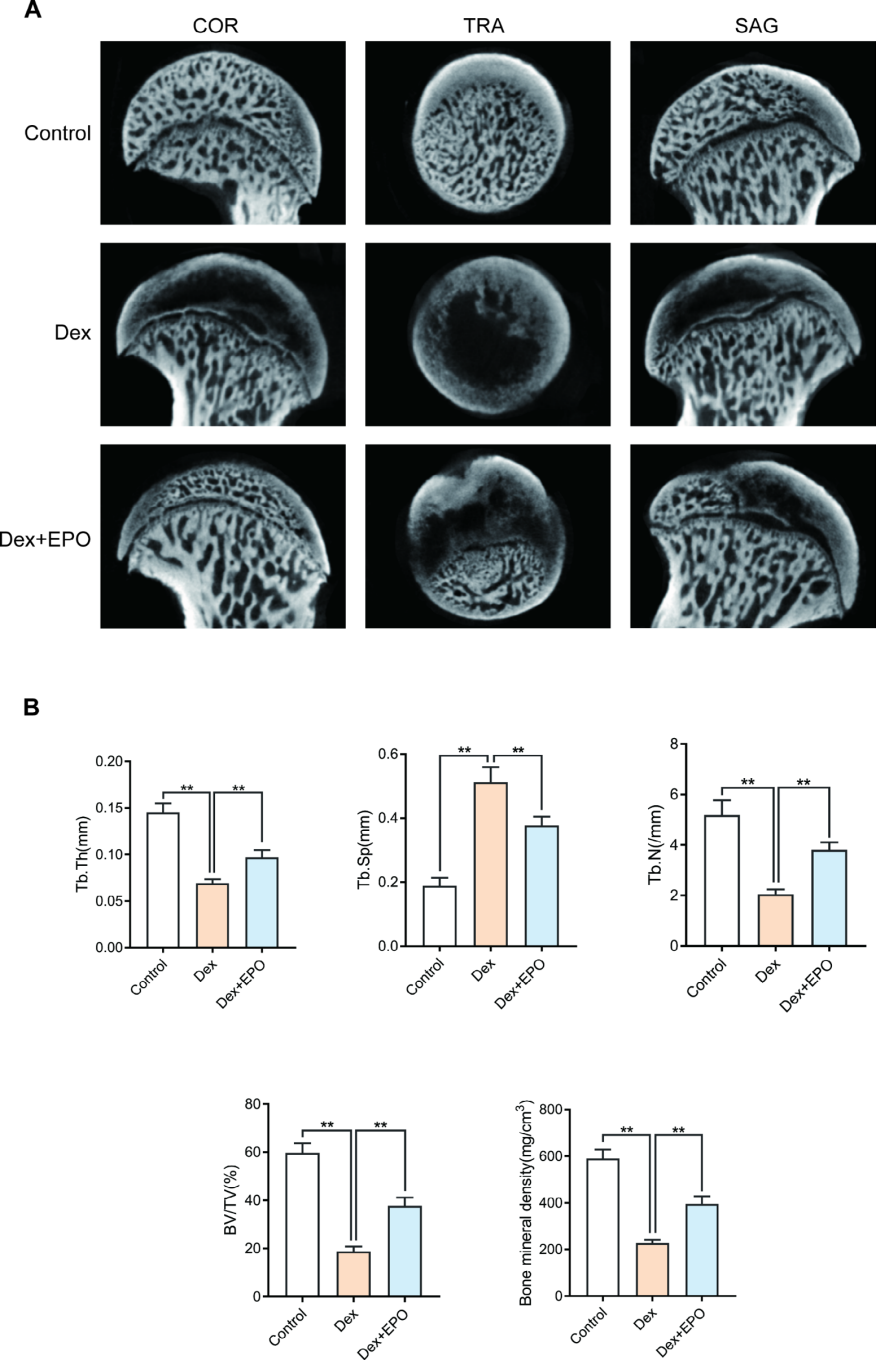
EPO prevented bone loss in the SANFH model of rats. In the various groups, the femoral head’s three plane images—coronal section (COR), sagittal section (SAG), and transverse section (TRA)—were rebuilt **(A)**. Quantitative analysis of micro-CT results. Bone mineral density (BMD), trabecular thickness (Tb.Th), trabecular separation (Tb.Sp), bone volume per tissue volume (BV/TV), and trabecular number (Tb.N)were analyzed in the different groups **(B)**. Values represent the averages ± S.D. Significant differences between different groups are indicated as *P < 0.05, **P < 0.01, n = 10

As shown in Fig. 7A, the control group’s subchondral trabecular bone had a normal distribution and was intact, with little osteonecrosis, as demonstrated by the Micro-CT. Images from the Dex group revealed a significantly damaged subchondral trabecular structure, an erratic low-density region, and a substantial loss of bone mass. However, EPO therapy can lessen femoral head necrosis and increase femoral head bone mass, indicating that EPO may cure SANFH. After the EPO treatment, the microstructural parameters such as bone mineral density (BMD), trabecular thickness (Tb. Th), trabecular separation (Tb. Sp), bone volume per tissue volume (BV/TV), and trabecular number (Tb. N) were significantly increased. Trabecular separation (Tb. Sp) was decreased when compared with the Dex group (Fig. 7B). In conclusion, dexamethasone-induced bone loss can be considerably halted by EPO.

## Discussion

In this study, we effectively induced SANFH in an experimental rat model through intramuscular administration of Dex, which led to prominent empty lacunae and extensive necrosis of bone marrow cells, as observed in H&E-stained slides. However, co-administration of EPO successfully attenuated the progression of these pathological changes, resulting in significant improvements in histological outcomes.

Acute spinal cord injury, viral infections, autoimmune disorders, shock, and other illnesses are all commonly treated with glucocorticoids (GCs), which also have potent anti-inflammatory and immunosuppressive properties. The extensive use of GCs will promote the induction of SANFH; joint replacement is usually the final treatment, but new pharmacological measures and the use of growth and differentiation factors to prevent and treat this disease have recently received widespread attention [25, 26]. Early research revealed that the mechanism of SANFH could be connected to defective cell differentiation, osteoporosis, microvascular coagulation, apoptosis, and arterial vascular injury. however apoptosis is currently thought to be a more common process [27, 28]. Cumulative evidence suggests that Dex and other glucocorticoids (GCs) contribute to the pathogenesis and development of nontraumatic osteonecrosis [29]. Apoptosis and osteoblast decrease are crucial for producing SANFH by glucocorticoids via glucocorticoid receptor activation [30, 31]. In a previous study, we provided direct evidence that the activation of STAT1-caspase 3 signaling during GC administration resulted in osteocyte apoptosis, which in turn contributed to the development of SANFH [14]. It has recently been shown that STAT1, a member of the STAT protein family, acts as a cytoplasmic transcription factor and activates several signal transduction pathways involved in various physiological and pathological responses. More proof indicates that STAT1 can cause apoptosis and obstruct bone fracture repair [32, 33]. Activated caspase 3 is a downstream target of STAT1 and is involved in several cellular processes, including the beginning of apoptosis, which results in the breakdown of cellular components [34]. Significant evidence has shown that STAT1 is crucial for controlling mitochondria-mediated apoptosis [13, 35]. In particular, the Bcl-2-BAX complex separates in response to the stimulation of apoptotic agents in osteocytes, causing a drop in Bcl-2 and a rise in BAX levels [36]. These proteins regulate mitochondrial membrane permeabilization, leading to the release of cytochrome C [37]. Caspase 9 is then activated, and Caspase 3 must be activated later [38]. In this study, Dex induced the progress of SANFH, as characterized by the increased expression of P-STAT1, BAX, cytochrome C, cleaved-caspase 9, and cleaved-caspase 3 along with decreased expression of Bcl-2. These results were confirmed by both histological examinations of femoral heads and immunofluorescence staining of osteoblasts, consistent with previous studies [13, 39].

The pleiotropic cytokine EPO is crucial for enhancing erythropoiesis. A variety of organs generates EPO and its receptor (EPO-R), and it is now understood that EPO serves physiological purposes other than erythrocyte production [40, 41]. Such as stimulating angiogenesis and cell regeneration, anti-inflammatory, anti-oxidation, anti-apoptosis and improving local microcirculation effects [42, 43]. As research on EPO and its receptors continues to expand, the application of their effects in clinical practice is increasingly evident. For example, its function of promoting erythropoiesis is used to treat various anemia and tumors [44]; its anti-inflammatory, anti-apoptosis, and other functions are used to treat the ischemia-reperfusion injury of myocardium [45]. It was reported that EPO can exert an anti-apoptotic effect by targeting and regulating STAT1 [20]. In the application of SANFH, EPO can effectively slow down the changes in bone morphology during SANFH and prevent the destruction of bone tissue structure, thus delaying or preventing SANFH [46]. By reducing the apoptosis rate of osteoblasts and osteoclasts, EPO can downregulate Caspase3 and upregulate the expression level of VEGF, thereby delaying the progression of the SANFH prevention process [21]. The precise mechanism by which EPO slows SANFH is yet unclear. In this work, we assessed the function of EPO in Dex-induced osteoblast apoptosis and its potential mechanism in vitro and in vivo.

In this research, we first examined the possible toxic concentration of EPO on osteoblasts using the CCK-8 assay. EPO dramatically reduced Dex-induced cell death, as shown by the LDH release test, in a concentration-dependent manner. Further confirmation of Dex-induced osteoblast cell death via apoptosis came from flow cytometric analysis, which showed that EPO could prevent Dex-induced apoptosis. Furthermore, we found that the expression levels of Cytochrome c and BAX and the protein production of Bc1-2 were significantly increased, which could be reversed by EPO-cotreated cells, suggesting that EPO acts as anti-apoptosis through the mitochondrial pathway.

STAT1, the first identified STAT family member, is essential for the regulation of caspase-3, and it was recently shown that its expression is significantly reduced in STAT1-knockout cells and is resistant to apoptosis, suggesting that STAT1 is involved in apoptosis [47]. Meanwhile, once STAT1 is activated, it can lead to upregulation of caspase3 expression, further promoting apoptosis [48, 49]. It has been reported that silencing or inhibiting STAT1 was shown to reduce apoptosis in the action of stimuli, all accompanied by altered caspase-3 expression levels, further indicating the close relationship between STAT1 and caspase3 in apoptosis [50, 51]. Moreover, it has also demonstrated that STAT1 and caspase3 are closely related to the development of SANFH [13, 14]. In this study, we found by Western imprinting and immunofluorescence assays that EPO inhibited Dex-induced apoptosis through the STAT1/caspase3 signaling pathway and that EPO may have potential therapeutic potential in treating SANFH and act in synergy with the effects of the STAT1 inhibitor Fludarabine. Furthermore, this effect may be achieved by inhibiting of STAT1 activity to mediate mitochondrial apoptosis. Immunohistochemistry testing revealed that EPO impacted caspase3 and STAT1 activation in vivo.

To comprehensively evaluate the protective function of EPO in vivo, we developed a SANFH animal model. In the Dex group, many rats exhibited hollow femoral heads, indicating significant bone necrosis and compromised blood flow, as observed through HE staining. Conversely, empty bone notch sockets were notably lower in the EPO group. Micro-CT results showed a substantially reduced SANFH severity in the EPO-treated group, with less severe trabecular destruction in the subchondral bone. Furthermore, EPO treatment led to improved microstructural parameters, including increased bone mineral density (BMD), trabecular number (Tb. N), and bone volume fraction (BV/TV), as well as decreased trabecular separation (Tb. Sp), compared to the Dex group. These findings suggest that EPO can potentially ameliorate SANFH in vivo by mitigating bone necrosis and preserving the structural integrity of the femoral head. While our study focused on an experimental animal model, it is essential to consider the clinical implications of these observations in human patients.

In clinical practice, SANFH often leads to debilitating symptoms and functional impairments in affected individuals. Current treatment options are limited, emphasizing the need for novel therapeutic approaches. Our study is promising preclinical outcomes of EPO warrant further investigation to ascertain its efficacy in human clinical trials. Several studies have already explored the clinical utility of EPO in related conditions [40, 41].

There are a few limitations to this study that should be mentioned. First of all, the sample size was somewhat tiny, and rats are known to exhibit unknown variability. Second, the mechanism of EPO in SANFH needs further investigation, such as whether EPO plays a role at the time of Dex interaction with its receptor and during intranuclear transfer following glucocorticoid receptor stimulation. Finally, whether data from Sprague-Dawley rats accurately reflect molecular changes in human SANFH is still being determined, though all target genes are evolutionarily conserved.

## Conclusion

**Fig. 8:**
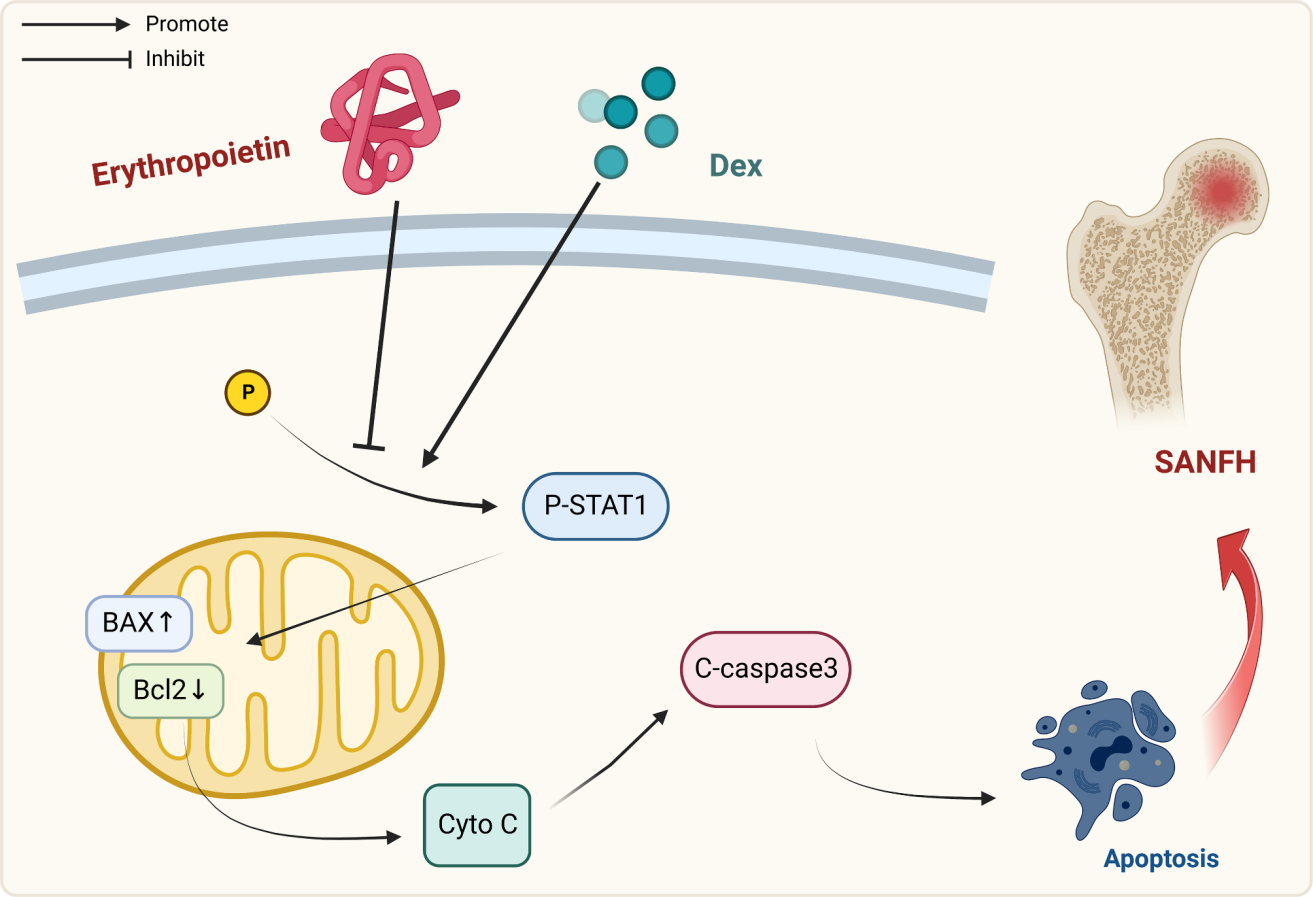
Schematic illustration of EPO protective effect against SANFH

Taken together, the data presented here suggest that EPO significantly inhibited the development of SANFH via inhibition of the STAT1-caspase 3 signaling pathway (Fig. 8). EPO is a therapeutic option for the management of SANFH.

### Electronic supplementary material

Below is the link to the electronic supplementary material.

Supplementary Material 1

## Data Availability

The data used to support the findings of this study are available from the corresponding author upon request.
