# Supplementary material for: Erythropoietin suppresses osteoblast apoptosis and ameliorates steroid-induced necrosis of the femoral head in rats by inhibition of STAT1-caspase 3 signaling pathway
Source: BMC Musculoskelet Disord. 2023 Nov 17;24:894. doi: 10.1186/s12891-023-07028-y (PMC10655348; doi:10.1186/s12891-023-07028-y)

P-STAT1 for Fig2

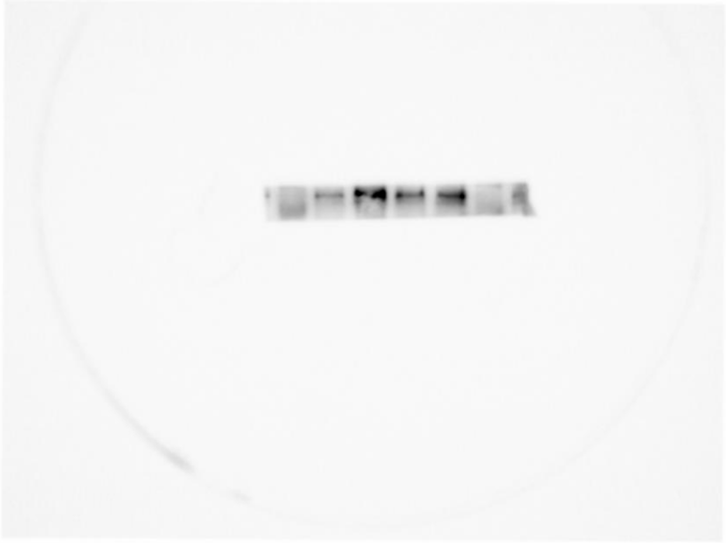

STAT1 for Fig2

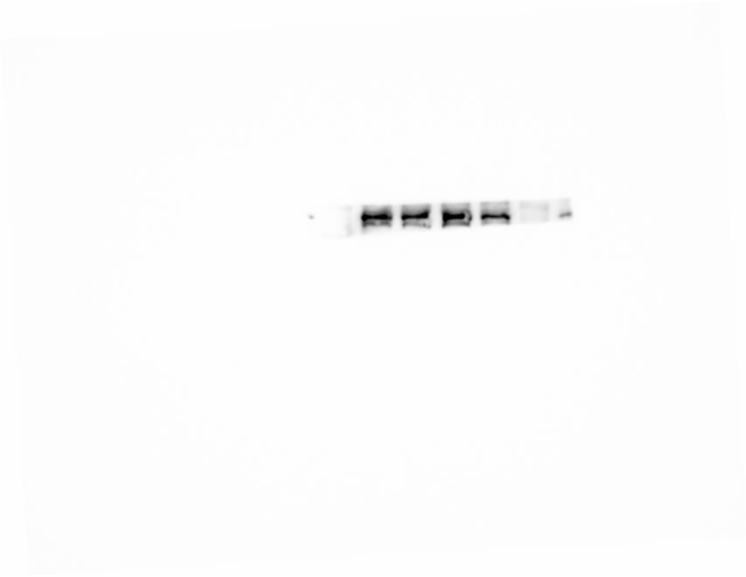

C-caspase 3 for Fig2

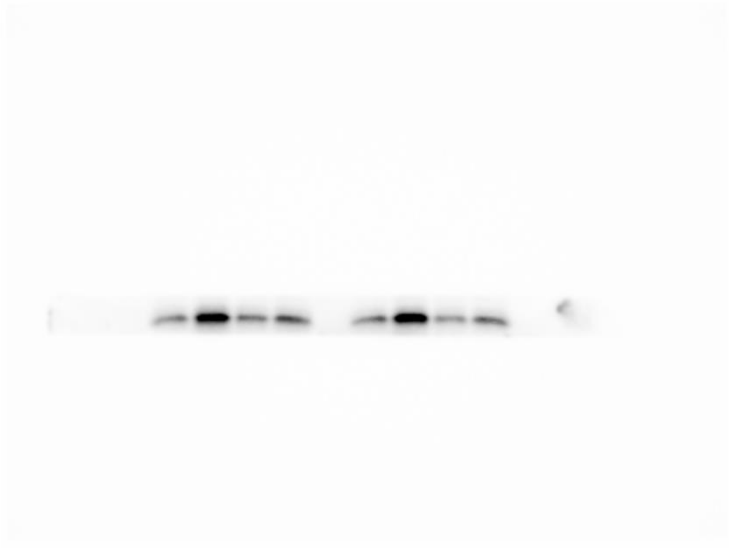

caspase 3 for Fig2

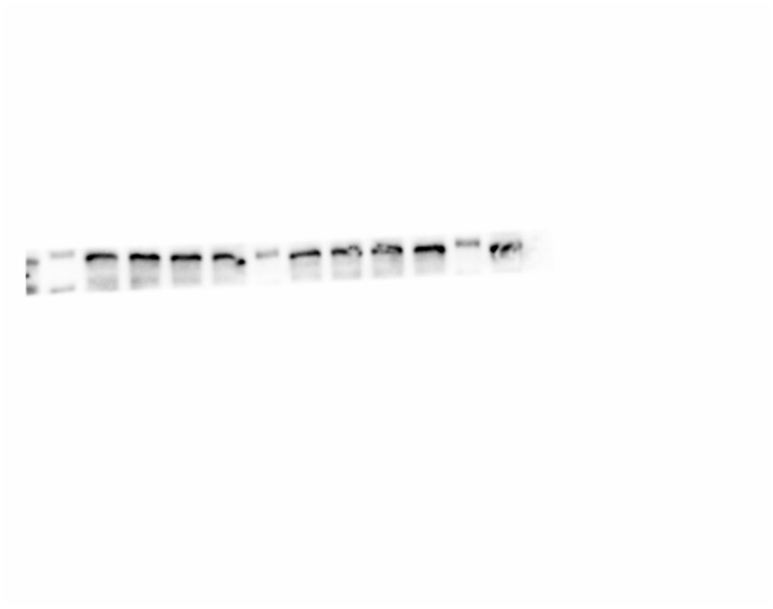

C-caspase 9 for Fig2

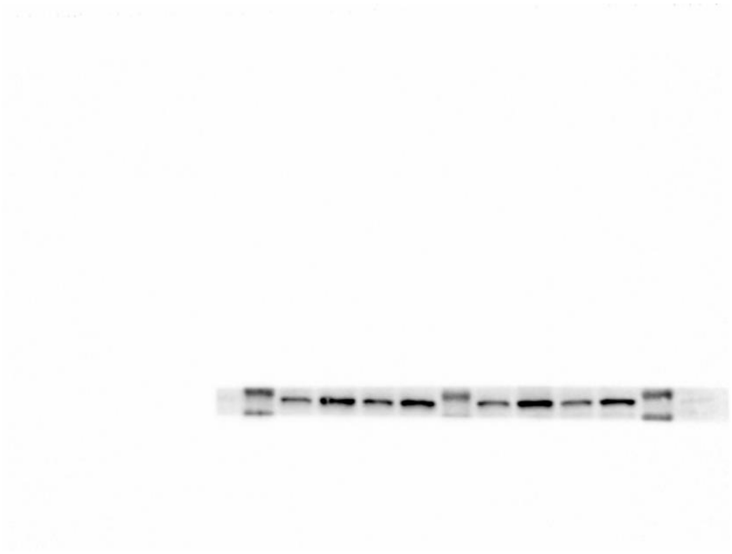

caspase 9 for Fig2

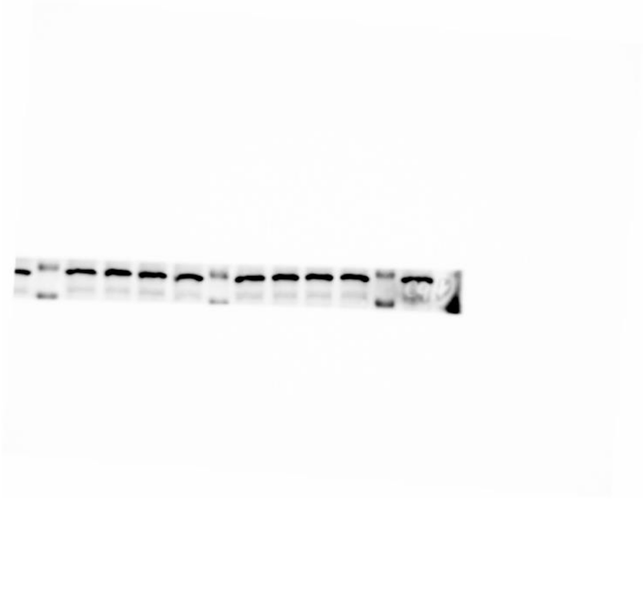

GAPDH for Fig2

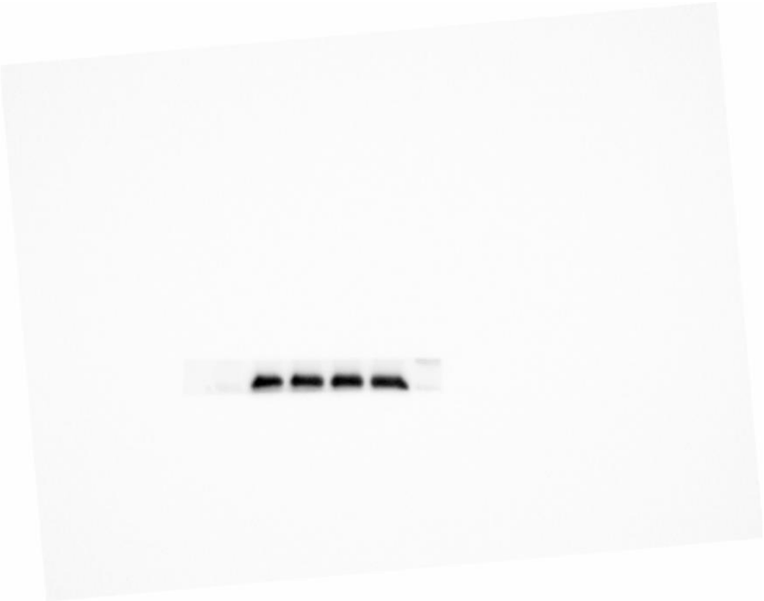

cytochrome C for Fig3

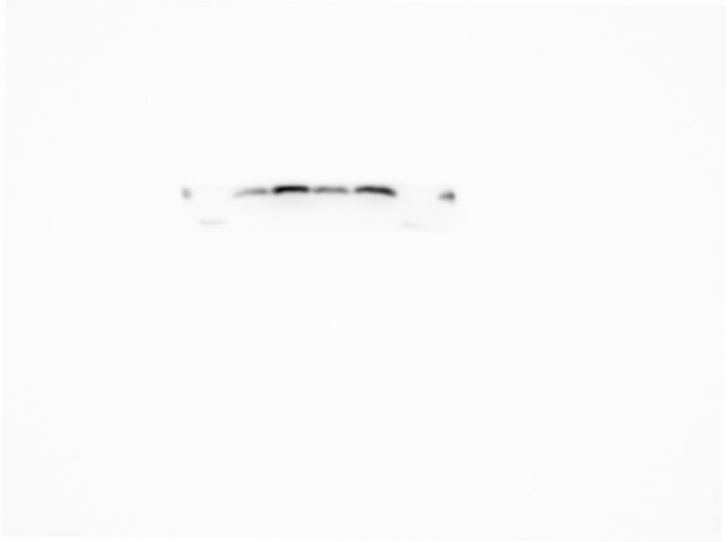

BAX for Fig3

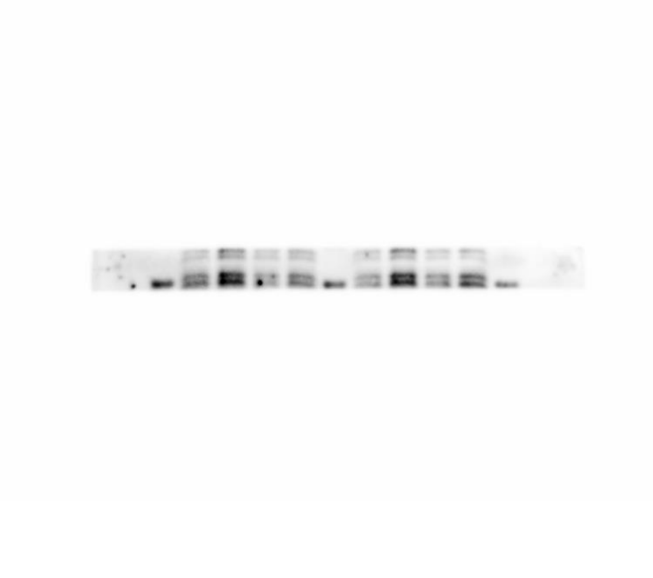

bcl 2 for Fig3

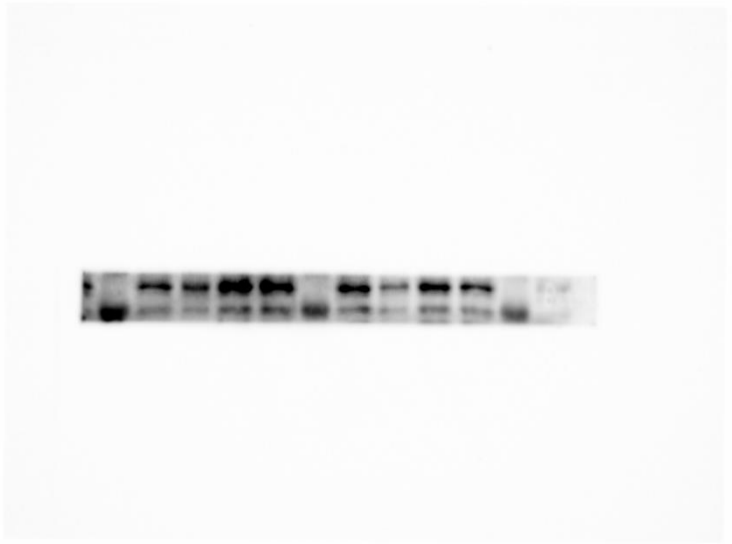

GAPDH for Fig3

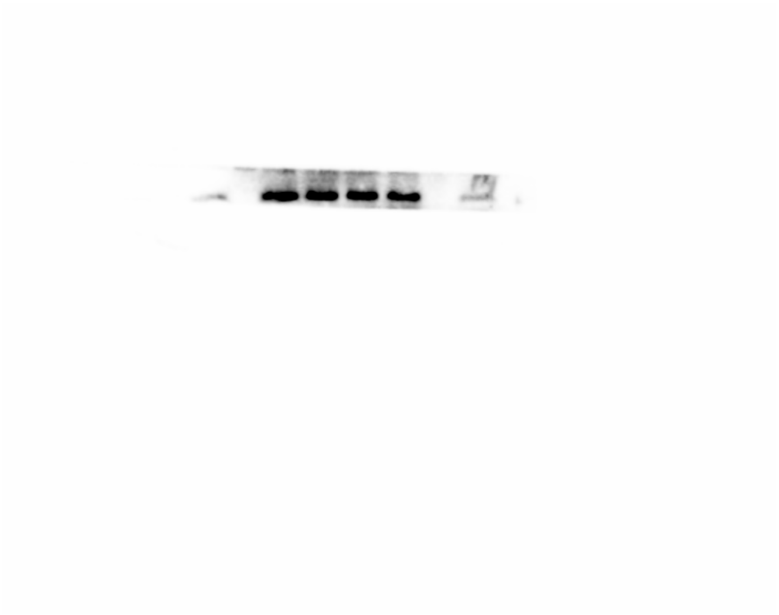

P-STAT1 for Fig4

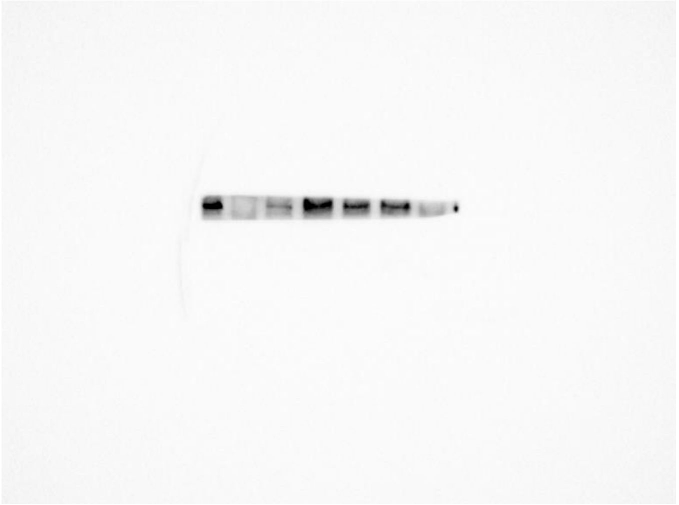

C-caspase 3 for Fig4

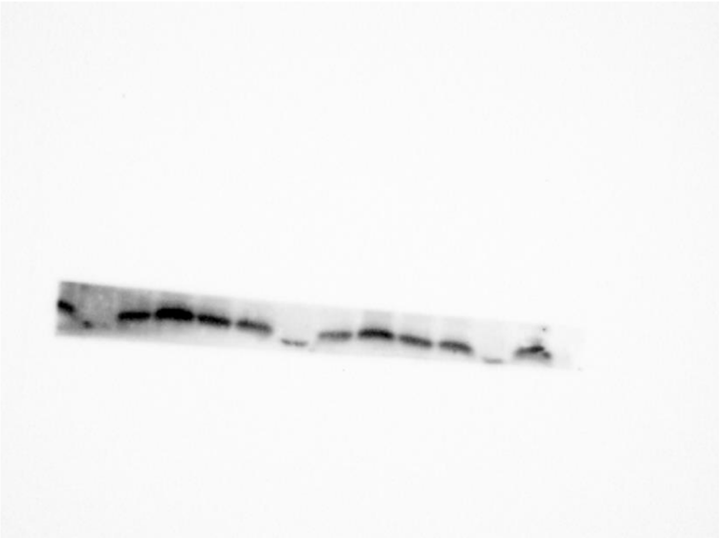

C-caspase 9 for Fig4

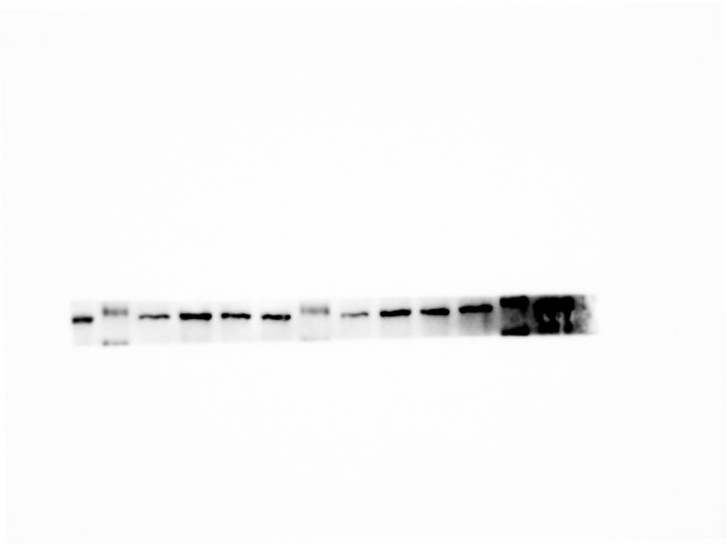

cytochrome C for Fig4

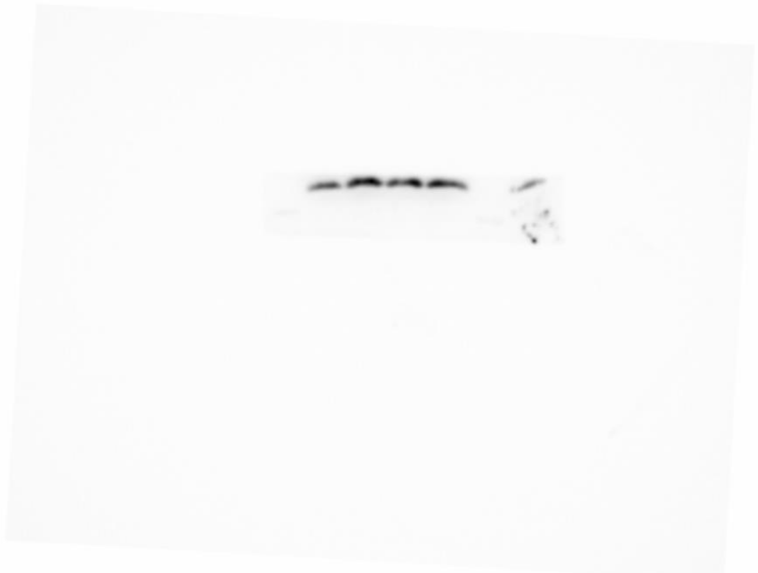

BAX for Fig4

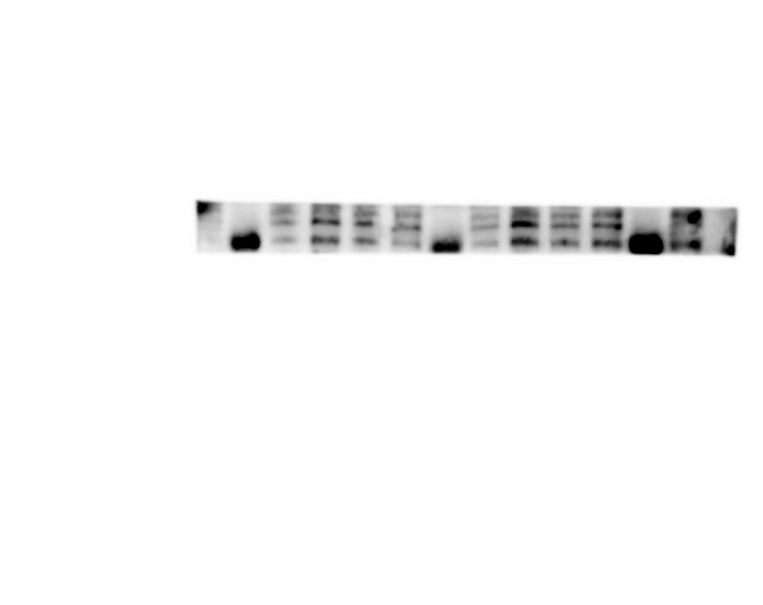

bcl 2 for Fig4

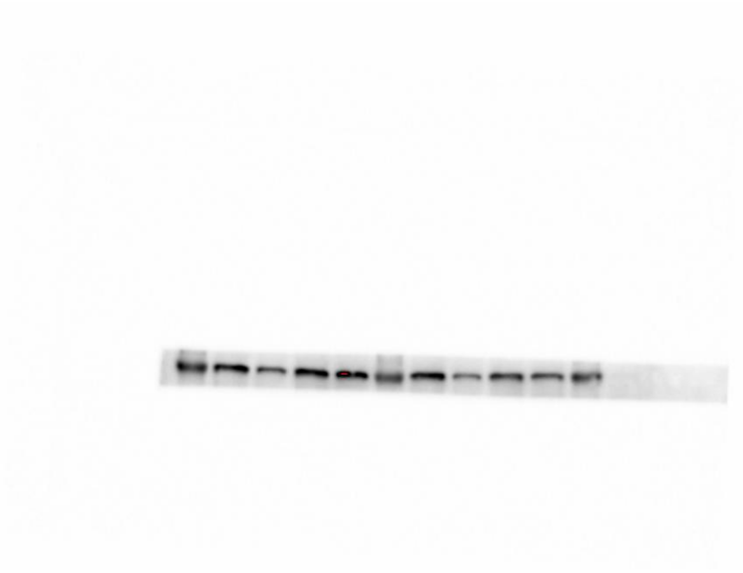

GAPDH for Fig4

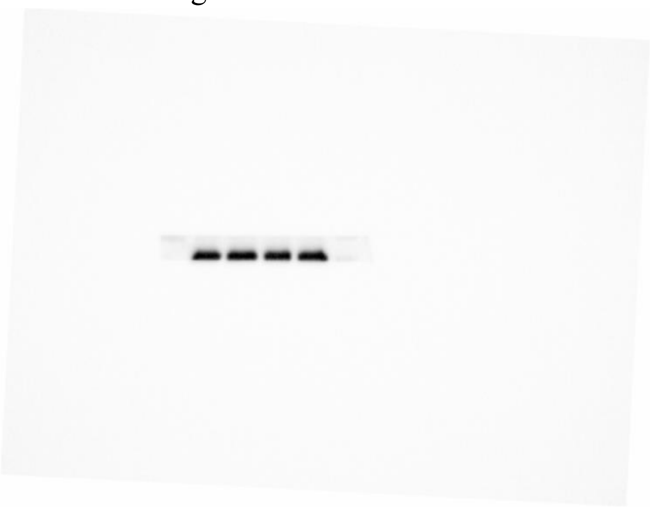

Supplement: Supplementary file 1 — Supplementary Material 1 [file 12891_2023_7028_MOESM1_ESM.pdf]
